# Comparison of the facial profile attractiveness in Class III borderline patients after surgical or compensatory orthodontic treatment

**DOI:** 10.4317/jced.56750

**Published:** 2020-04-01

**Authors:** Julie-Heide-Miyazaki Watanabe, Francisco Fitarelli, Daniel-Salvatore de Freitas, Rodrigo-Hermont Cançado, Renata-Cristina-Gobbi de Oliveira, Ricardo-Cesar-Gobbi de Oliveira, Fabricio-Pinelli Valarelli, Karina-Maria-Salvatore Freitas

**Affiliations:** 1 DDS, MSc. Orthodontic Graduate Student. Department of Orthodontics. UNINGÁ University Center. Maringá, Brazil; 2 DDS, MSc, PhD. Professor. Freitas Dentistry Institute. Bauru, Brazil; 3 DDS, MSc, PhD. Professor. Department of Orthodontics. UNINGÁ University Center. Maringá, Brazil

## Abstract

**Background:**

This study aimed to compare the facial profile attractiveness of Class III borderline patients after surgical or compensatory orthodontic treatment.

**Material and Methods:**

The sample consisted of 60 borderline Class III malocclusion patients, divided into two groups: Group 1 (Surgical): 30 patients (16 male; 14 female) treated with orthodontic fixed appliances and bimaxillary orthognathic surgery. Mean initial age was 20.05 years (s.d.=2.40) and mean treatment time was 2.23 years (s.d.=0.82). Group 2 (Compensatory): 30 patients (13 male; 17 female) treated compensatorily with fixed appliances and Class III elastics. Mean initial age was 18.53 years (s.d.=4.35) and mean treatment time was 2.08 years (s.d.=0.67). Silhouettes of the facial profile were constructed obtained from the pretreatment and posttreatment lateral cephalograms and evaluated by orthodontists (N=41, 22 females and 19 males, mean age of 35.65 years), assigning scores from 1 (least attractive) to 10 (most attractive). Intergroup comparison of profile attractiveness was performed by Mann-Whitney test. For intragroup comparison of initial and final stages, the Wilcoxon test was used.

**Results:**

At initial stage, the compensatory group presented a statistically significant greater attractiveness of the profile than the surgical group. With treatment, the surgical group presented significantly more improvement in facial profile than the compensatory group. At the final stage, profile attractiveness of surgical and compensatory groups was similar.

**Conclusions:**

The facial profile attractiveness is similar in Class III patients after orthognathic surgery or compensatory orthodontic treatment. However, surgery provided more improvement in profile attractiveness than the compensatory treatment in Class III patients.

** Key words:**Malocclusion, angle Class III, orthognathic surgery, corrective orthodontics.

## Introduction

The parameters of beauty and facial attractiveness have considerable influence on the population, since esthetic standards are seen as an important factor for social acceptance. More than 70% of parents believe that their children will become more attractive, socially accepted and successful in their professional life after orthodontic treatment (1). Orthodontics and oral and maxillofacial surgery are dental specialties that allow corrections of positioning, functional, dentofacial and alterations, with improvements in terms of facial aesthetics and attractiveness (2).

Malocclusion is known as a factor that negatively interferes in facial and smile attractiveness; the Class III malocclusion is the one that shows the greatest impairment of facial esthetics (3). This malocclusion can be treated orthopedically with maxillary expansion and reverse traction during the growth phase, but after the pubertal growth phase, the treatment options are compensatory orthodontic treatment or orthognathic surgery (4-6).

Orthognathic surgery can correct skeletal discrepancies, modifying the skeletal pattern of the patient and producing remarkable profile changes (2). In some cases, even though malocclusion can be corrected with compensatory orthodontics and an ideal occlusion is achieved, a surgical treatment plan is suggested in attempt to improve the facial profile esthetics.

The facial attractiveness is generally the deciding element in treatment planning of borderline patients who can be treated with compensatory orthodontic treatment or orthognathic surgery (7). In Class III patients, the facial profile and skeletal discrepancy are sometimes the main focus of the patients, and in these cases the profile improvement should be the major goal in treatment outcomes (8).

Johnston *et al.* (9) found that profiles with normal SNB angle are the most attractive; prominent mandibles were more attractive than deficient ones. Phillips, Trentini and Douzartzidis (10) compared orthodontic with surgical treatment and found that the orthodontics group was more attractive at the beginning and at the end of orthodontic treatment than the surgically treated profiles. On the other hand, surgical treatment showed to improve more the facial attractiveness, whereas the orthodontic treatment only maintained the initial attractiveness of the patients.

Adamian (11) compared the profile attractiveness of borderline Class III cases treated with surgery or orthodontically and found that surgery or camouflage treatment provides similar esthetic improvement in profile attractiveness in borderline Class III surgical/orthodontic patients. However, she used modified profile image showing masking of the eyes, eyebrows and hair and not the profile silhouettes (11).

The objective of this study was to compare the facial profile attractiveness of borderline Class III patients treated with surgical or compensatory orthodontic treatment.

## Material and Methods

-Material

This retrospective study was approved by the Ethics Committee in Human Research of the UNINGA University Center, Maringá, Brazil, and all patients signed an informed consent.

The sample size calculation was based on a significance level alpha of 5% and beta of 20% to achieve a power of the test of 80% to detect a minimum difference of 0.81 points for the score of profile attractiveness, with a standard deviation of 1.1 (10). The sample size calculation showed the need for 30 subjects in each group.

Inclusion criteria for sample selection were: borderline Class III patients with skeletal discrepancy of the facial profile; Class III malocclusion with at least half-cusp Class III molar relationship at the beginning of treatment; ANB of -1° or less; initial treatment planning including both options of compensatory or surgical-orthodontic treatment; all teeth present up to the first molars; no agenesis or supernumerary teeth; no previous orthodontic or orthopedic treatment.

The sample consisted of 60 borderline Class III malocclusion patients treated orthodontically with orthognathic surgery or compensatorily, divided into two groups:

Group 1 (Surgical): 30 patients (20 male; 15 female) treated with orthodontic fixed appliances and orthognathic surgery, obtained from the files of the from the files of the Freitas Dentistry Institute, Bauru, Brazil. Mean initial age was 20.05 years (s.d.=2.40), mean final age was 22.28 years (s.d.=3.18) and mean treatment time was 2.23 years (s.d.=0.82). Initial severity of the Class III malocclusion was: 6 patients with full-cusp Class III, 13 with ¾-cusp, 11 with half-cusp Class III malocclusion. Orthognathic surgery of all of the patients included combined maxillary advancement and mandibular setback. Surgical treatment planning was made with Dolphin Imaging software (Dolphin Imaging & Management Solutions version 11.5; Chatsworth, Calif, USA). The same surgeon (DSF) performed the orthognathic surgery of all patients in hospital environment with general anesthesia.

Group 2 (Compensatory): 30 patients (16 male; 14 female) treated compensatorily with fixed appliances and Class III elastics, obtained from the files of the from the files of the IOPG, Bauru, Brazil. Mean initial age was 18.53 years (s.d.=4.35), mean final age was 20.61 years (s.d.=3.99) and mean treatment time was 2.08 years (s.d.=0.67). Initial severity of the Class III malocclusion was: 3 patients with full-cusp Class III, 12 with ¾-cusp, 15 with half-cusp Class III malocclusion. The mechanics used for Class III compensatory treatment included fixed preadjusted appliance (Class III Biofunctional prescription, slot 0.022”x0.030”, Morelli, Sorocaba, São Paulo, Brazil). Leveling and alignment was performed with 0.014”, 0.016” and 0.018” Nitinol and 0.020” and 0.019x0.025” stainless steel archwires. The main mechanics for Class III correction was the use of heavy 3/16” Class III intermaxillary elastics. The Biofunctional prescription of fixed appliances includes lingual crown torque on the maxillary anterior teeth and labial crown torque on the mandibular anterior teeth to counteract the Class III elastics (4-6).

**Figure 1:**
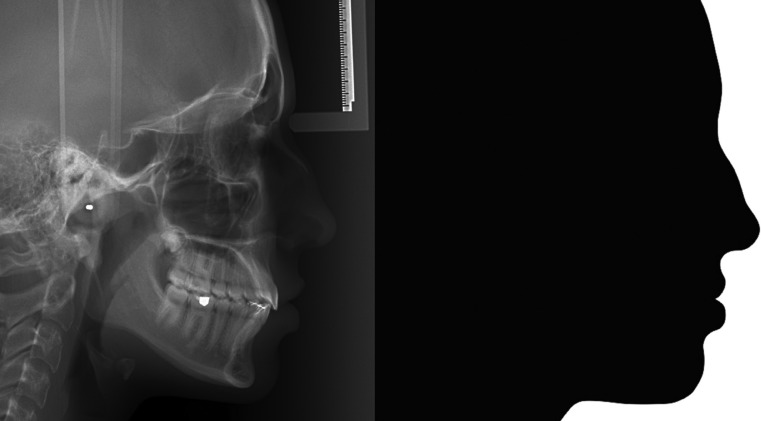
Facial profile silhouette obtained from the lateral cephalogram.

The lateral cephalograms from pretreatment and posttreatment were used. From these cephalograms, silhouettes of the facial profile were constructed (Fig. 1) and evaluated by expert orthodontists (N=41, 22 females and 19 males, mean age of 35.65 years). The images of the silhouettes were cropped in the Microsoft Office Picture Manager program, with 3x4 ratio, in portrait format. After randomization, imagens were sent to a site (google forms) and a link was sent to the orthodontists to perform the evaluations. The evaluators assigned scores for each facial profile from 1 (least attractive) to 10 (most attractive). They could look at the images for as long as they wish and change the scores before submitting the form.

-Error study

The reliability and precision of the methodology were verified by the Kappa coefficient in 20 randomly selected silhouettes, in which the attractiveness was reevaluated for 12 randomly selected orthodontists within a month interval. The Kappa coefficient was 0.89, which is considered as an excellent agreement (12).

-Statistical analysis

Normality of the data was verified by Shapiro-Wilk test. Since data did not show normal distribution, nonparametric tests were used.

Intergroup comparisons of sex distribution and severity of Class III malocclusion were performed with chi-square tests and initial and final ages and treatment times were compared by independent t tests.

Intergroup comparison of profile attractiveness was performed by Mann-Whitney test. For intragroup comparison of initial and final stages, the Wilcoxon test was used.

All tests were performed using the software Statistica (Statistica for Windows, version 7.0, Statsoft, Tulsa, Okla, USA) and results were considered significant for *P*<0.05.

## Results

Groups were comparable regarding initial and final ages, treatment time, sex distribution and severity of Class III malocclusion (Table 1).

**Table 1:**
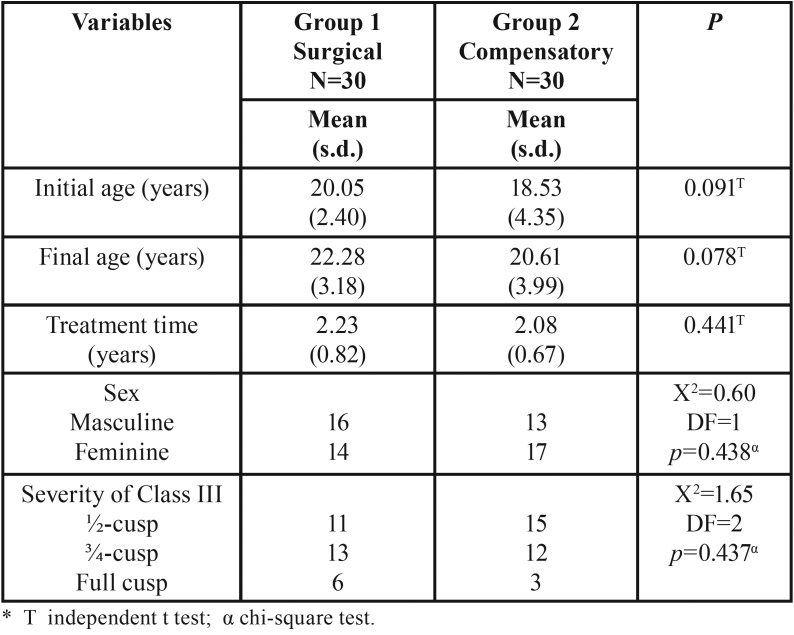
Results of intergroup comparability of initial and final ages, treatment time, sex distribution and severity of Class III malocclusion.

At initial stage, surgical group presented a statistically significant lesser attractive profile than the compensatory group (Table 2). With treatment, both surgical and compensatory groups presented a statistically significant improvement of the facial profile attractiveness (Table 3), but the surgical group presented significantly more improvement than the compensatory group (Table 2). At the final stage, after treatment, the two groups presented similar facial profile attractiveness (Table 2).

**Table 2:**
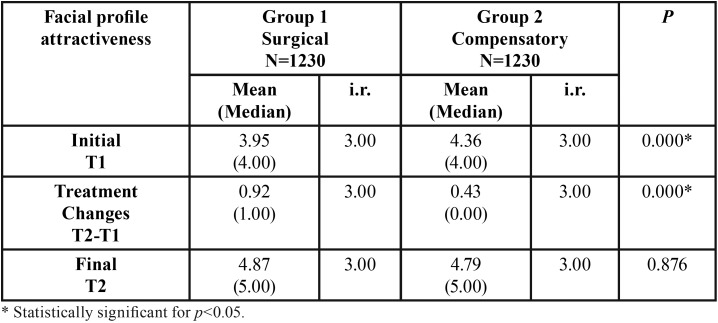
Results of intergroup comparison of facial profile attractiveness at initial and final stages and treatment changes (Mann-Whitney nonparametric test).

**Table 3:**
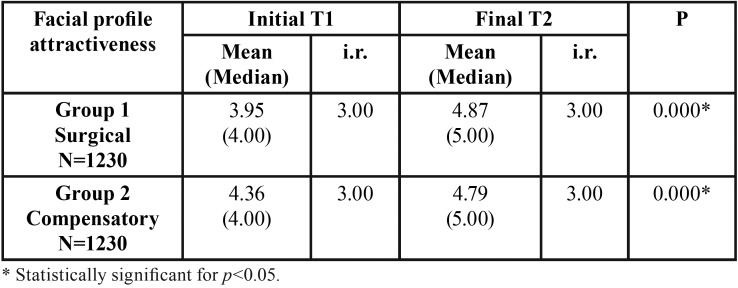
Results of intragroup comparison of facial profile attractiveness at initial and final stages (Wilcoxon nonparametric test).

## Discussion

The crescent interest in facial esthetics increased the search for orthodontic treatment and led orthodontists and patients to seek for treatments that result in better facial esthetics and not just an ideal occlusion. The silhouettes area a great method of assessing the facial profile esthetics because it eliminates confounding factors that influence the attractiveness, such as age and sex of the patient and color of the skin, hair and eyes (13-15).

Patients with ¼-cusp Class III malocclusions and less than -1° of ANB angle were excluded, attempting to match the samples of both groups. Usually, less severe Class III cases are more likely to be treated with compensatory orthodontics and more severe cases with orthognathic surgery. In this way, we eliminate the less severe cases trying to reach comparability of the malocclusion severity between the groups, since it is known that the profile attractiveness is related to the severity of the malocclusion (9).

This methodology of evaluation of the facial silhouettes was previously used in several studies and, besides subjective, is considered reliable and reproducible (10,11,13,14,16).

At the beginning, orthodontic compensatory group presented a significantly more attractive profile than the surgical group (Table 2). This is probably because the surgical group had a Class III molar relationship slightly more severe than the compensatory group, besides not showing a statistically significant difference (Table 1). Phillips, Trentini and Douzartzidis (10) also found that the camouflage Class III group was more attractive at the beginning, but they did not match the severity of the malocclusion between the groups. Also, Georgalis and Woods (17) found that, before treatment, the surgical group demonstrated, on average, a more severe skeletal discrepancy and increased dental compensations, compared with the orthodontically camouflaged group.

Both compensatory orthodontic treatment and orthognathic surgery improved significantly the facial profile attractiveness; but the orthognathic surgery improved more ( Table 2, Table 3). Phillips, Trentini and Douzartzidis (10) found no significant improvement in orthodontics group, and a significant improve for the surgical group. However, if we look at some cephalometric measures, it can be noticed that the surgical group presented greater severity at the beginning and was more corrected with treatment, justifying the results.

Adamian (11) also compared borderline Class III cases and found that surgery and camouflage treatment provide similar esthetic improvement in profile attractiveness. However, she used a modified profile image showing masking of the eyes, eyebrows and hair and not the profile silhouettes, as we used (11).

At the final evaluation, the compensatory and surgical groups presented similar facial profile attractiveness (Table 2). Phillips, Trentini and Douzartzidis (10) found that orthodontic camouflage treatment resulted in a more attractive profile than the orthognathic surgery; however, the lack of match in the groups that were compared impair the results.

Camouflage treatment of the Class III malocclusion produces proclination of maxillary incisors, retrusion of mandibular incisors, and downward and backward rotation of the mandible (18) in order to compensate for an underlying maxillomandibular discrepancy (2). Orthognathic surgery can modify the skeletal pattern and produce remarkable facial profile changes (2). This is corroborated by the present study results, that showed more improvement of the profile attractiveness with surgical treatment.

When Class III borderline patients are considered for treatment, the planning should focus on the patients’ chief complaint and the facial profile involvement (8), and, in conjunct with the orthodontist and oral surgeon, to choose the best treatment option for each case. Surgical-orthodontic treatment involves more correction of the facial profile than compensatory orthodontic treatment alone, but when profile is not the main complaint of the patient, the compensatory treatment can produce satisfactory occlusal and acceptable facial results.

## Conclusions

The facial profile attractiveness is similar in Class III patients after orthognathic surgery or compensatory orthodontic treatment. However, surgery provided more improvement in profile attractiveness than the compensatory treatment in Class III patients.
